# Antibacterial activity and phytochemical screening of traditional medicinal plants most preferred for treating infectious diseases in Habru District, North Wollo Zone, Amhara Region, Ethiopia

**DOI:** 10.1371/journal.pone.0300060

**Published:** 2024-03-05

**Authors:** Mulugeta Alemu, Ermias Lulekal, Zemede Asfaw, Bikila Warkineh, Asfaw Debella, Abiy Abebe, Sileshi Degu, Eyob Debebe

**Affiliations:** 1 College of Natural and Computational Sciences, Department of Plant Biology and Biodiversity Management, Addis Ababa University, Addis Ababa, Ethiopia; 2 Nefas Silk Polytechnic College, Department of Urban Agriculture, Addis Ababa, Ethiopia; 3 Armauer Hansen Research Institute, Traditional and Modern Medicine Research and Development Directorate, Addis Ababa, Ethiopia; University of Jeddah, SAUDI ARABIA

## Abstract

Ethiopia’s healthcare system relies on traditional medicinal practices that use medicinal plants to treat human and livestock ailments. However, the lack of empirical validation regarding the efficacy of these treatments against specific infectious diseases necessitates rigorous scientific investigations. The objective of this study was to investigate the antibacterial activity and phytochemical screening on five selected medicinal plant species, namely *Solanum somalense* Franchet., *Verbascum sinaiticum* Benth., *Rumex nervosus* Vahl, *Withania somnifera* (L.) Dunal and *Calpurnia aurea* (Ait.) Benth. The plants were first identified jointly with local informants and later considering mainly their high informant consensus and fidelity level values for their efficacy in treating infectious diseases in the area. Ethanol and aqueous extracts were prepared from the plant materials, and their antibacterial activities were evaluated against standard bacterial strains, representing both gram-positive and gram-negative types. To assess the antibacterial activity of the extracts, the minimum inhibitory concentration (MIC) was determined using the broth dilution method. Additionally, phytochemical screening was performed using standard qualitative tests to identify various secondary metabolites. The results indicated antibacterial efficacy in the ethanol extracts of *S*. *somalense*, *W*. *somnifera*, and *C*. *aurea* against particular bacterial strains (*S*. *somalense* against *S*. *agalactiae* with MIC of 1.5 mg/mL; *W*. *somnifera* against *S*. *aureus* and *E*. *coli*, with MIC of 2 mg/mL; *C*. *aurea* against *E*. *coli* and *K*. *pneumoniae*, with MICs of 3 mg/mL and 3.5 mg/mL, respectively). The results of the phytochemical screening indicated the presence of steroids, alkaloids, flavonoids, saponins, and terpenoids. The selected medicinal plants demonstrated promising antibacterial activity against certain bacterial strains. The current findings support the long-standing claim of the traditional medical system of the study area for their continued use of these plants in their treatment of infectious diseases. Further investigation is required to isolate the responsible active compounds and characterize the constituents and description of their antibacterial effect for possible use in areas where these infectious diseases are major health problems.

## Introduction

Despite significant advancements in modern medicine, microbial diseases persist as substantial global threats [1]. Acknowledging this, the World Health Organization emphasizes herbal medicine as a primary healthcare modality in numerous developing nations [2]. Globally, approximately 80% of the population relies on medicinal plants for disease treatment, with a more pronounced prevalence in African nations [2]. Ethiopia, recognized for its rich biodiversity and profound traditional knowledge of medicinal plants, significantly incorporates these resources into its healthcare system. Recent research conducted by [3, 4] reaffirms that around 80% of the population and 90% of livestock in Ethiopia depend on traditional medicine, attributing medicinal plants as the cornerstone of these practices and emphasizing their role in treating various ailments, including bruised ulcer, malaria, diabetes, cancer, and infectious diseases.

Plants are vital resources for drug discovery due to their extensive historical use in traditional medicine [5]. The Food and Agriculture Organization report indicates that at least 25% of pharmaceutical drugs in modern pharmacopoeia are plant-derived, with numerous others being synthetic analogues derived from plant prototype compounds [6]. Throughout history, natural products from plants have served as medicine [7, 8] displaying a diverse array of bioactive compounds effective against various infectious agents [9]. Vital methods for assessing bioactive properties and identifying compounds from plant extracts include antibacterial activity testing and phytochemical screening [10, 11].

Previous research has explored the antibacterial and phytochemical properties of *Verbascum sinaiticum* Benth. (Scrophulariaceae), *Rumex nervosus* Vahl (Polygonaceae), *Withania somnifera* (L.) Dunal (Solanaceae) and *Calpurnia aurea* (Ait.) Benth. (Fabaceae) [12–18]. However, there is no investigation report available concerning the antibacterial and phytochemical properties of *Solanum somalense* Franchet. (Solanaceae). Despite the existing research on these plants, there is still a need for further investigation into their antibacterial and phytochemical properties. In developing countries like Ethiopia, where access to modern drugs is constrained, the impact of microbial diseases is notably pronounced [19]. The Habru District, situated in the North Wollo Zone of the Amhara Regional State, mainly relies on traditional medicine within its primary healthcare system. In spite of its significance, the botanical diversity and ethnobotanical practices in this region lack comprehensive study and documentation. Consequently, this study aims to evaluate the antibacterial activity and phytochemical constituents of select medicinal plants, considering the degree of informants’ consensus and fidelity level values for treating infectious diseases.

## Materials and methods

### Selection of medicinal plants and plant material collection and preparation

Ethnobotanical methods were applied as recommended by [20, 21] and ethnobotanical data on herbal use and preparation of the medicinal plants were collected by interviewing general informants, herbalists and key informants. The ethnobotanical field study was carried out from November 26/2022 –December 14/2022 in Habru District. This study encompassed a total of 388 informants (250 males and 138 females) from all 13 kebeles within Habru District. The selection of these informants followed systematic random and purposive sampling methods, including peer recommendations, as described by [20]. Qualitative and quantitative ethnobotanical data were collected from informants through a pre-prepared semi-structured interview method, as described by [20–22]. In the broader context of this study, techniques such as group discussions, semi-structured interviews, guided field walks, market surveys, preference ranking, and direct matrix ranking were employed [20]. Additionally, a total of 13 focus group discussions were carried out, one at the district level and 12 at selected kebeles of the district. In each focus group discussion key informants, traditional healers, elders, kebele and district administrative officials from natural resource and forest protection offices, and agricultural and rural development offices were involved to amplify insights into medicinal plant knowledge at the community level and to corroborate information obtained through semi-structured interviews. Along with collection of ethnobotanical data, voucher specimens of traditional medicinal plant species were collected, taxonomically identified, authenticated, confirmed by a taxonomic expert and deposited at the National Herbarium of Ethiopia, AAU. From among the plants recorded, five species (Table 1) that were frequently recommended by traditional herbalists and knowledgeable elders for their efficacy in treating infectious diseases, and have relatively higher informant consensus and fidelity level values were selected for antibacterial activity and phytochemical constituents [5].

**Table 1:** Ethnobotanical data on medicinal plants selected for antibacterial tests and phytochemical screening.

Scientific name	Family name	Local name (Amharic)	Parts used	Locally used in the study area to treat	FL value (%)	Frequencies of citations	Voucher no.
*Solanum somalense* Franchet.	Solanaceae	Yeshehochu Kitel /Shejerete Jin	Leaves	Headache, **diarrhoea**, evil eyes, febrile disease, fever, evil spirits, swelling, toothache	91.3	39	MA114
*Verbascum sinaiticum* Benth.	Scrophulariaceae	Yahya Jero	Leaves	Febrile disease, **wound**	85.7	23	MA120
*Rumex nervosus* Vahl	Polygonaceae	Embacho	Leaves, root	Warts, **wound**, fire burn diarrhoea	72.2	20	MA104
*Withania somnifera* (L.) Dunal	Solanaceae	Ede-buda/Gizawa	Leaves, root	Headache, **toothache**, evil eyes, syphilis, gonorrhoea, febrile disease, fever, evil spirits, swelling	81.8	26	MA124
*Calpurnia aurea* (Ait.) Benth.	Fabaceae	Digita	Leaves and root	**Diarrhoea**, snake bite, excessive bleeding after birth	75	16	MA16

**Remark:** The FL value % in the table represents the importance of each species in treating specific diseases, as indicated by the bold entries in the disease category column. A higher FL value signifies a greater significance of the respective species in the treatment of the mentioned ailments in the study area.

In a recent study, we identified *Solanum somalense*, *Verbascum sinaiticum*, *Rumex nervosus*, *Withania somnifera and Calpurnia aurea* as key medicinal plants within the Habru District, Amhara Region, Ethiopia [23]. Building upon this foundational research, fresh leaves of these selected species were collected for further analysis. The collected plant material was dried at room temperature and using solar drier (35–40°C), and the plant cutlets were milled to powder. The powder was accurately weighed using an electronic weighing balance, then packed into polyethylene bags to prevent air entry and contamination. Finally, the bags were stored in a securely closed container, appropriately labeled, to facilitate for further extraction processes.

#### Preparation of extracts

The dried plant material of each species was finely ground and the powder was extracted by maceration while shaking using orbital shaker successively until exhaustion, 80% ethanol (500 mL) and aqueous/distilled water were used as extraction solvents [24, 25]. The 80% ethanol extracts from each plants were subsequently filtered using Whatman filter paper followed by concentration under reduced pressure (vacuum) by using a rotary evaporator (R-300 Buchi, Switzerland) *in vacuo* at 40°C to give gummy residue while the aqueous extracts of each plants were lyophilized to yield of amorphous powder [1, 25]. Finally, the concentrated extracts were dried and kept in a refrigerator until used for the experiment.

#### Microorganisms

The antibacterial activity of leaf extracts of the selected MPs was evaluated against selected standard bacterial strains of the American Type Culture Collection (ATCC). The bacterial strains selected were representative of both classes of Gram-positive and Gram-negative bacteria. The tested gram-positive bacterial strains were *Staphylococcus aureus* (ATCC 25923), *Streptococcus agalactiae* (ATCC 12386), *Staphylococcus epidermidis* (ATCC 12228), *Enterococcus faecalis* (ATCC 29212) and those of the gram-negative bacterial strains included *Proteus mirabilis* (ATCC 35659), *Salmonella typhimurium* (ATCC 13311), *Klebsiella pneumoniae* (ATCC 700603), *Escherichia coli* (ATCC 25922) and *Shigella flexneri* (ATCC 12022). The microorganisms were maintained at the Traditional and Modern Medicine Research Directorate (TMMRD) of Armauer Hansen Research Institute (AHRI) microbiology laboratory on Triptosoya + 20% glycerol broth at -78°C. Finally, the selected bacterial strains were cultivated using Mueller-Hinton broth [26].

#### Ethical consideration

Ethical considerations were carefully addressed throughout the study to ensure the protection of community knowledge and the rights of the participants. Official permission from relevant authorities and local stakeholders (Addis Ababa University, Department of Plant Biology and Biodiversity Management, Armauer Hansen Research Institute, and Habru District Administration) was obtained to conduct the research in the study area. Prior to the commencement of interviews for ethnobotanical data collection, informed consent was obtained from each participant. This process involved a thorough explanation of the study’s objectives and an assurance of judicious use of the information gathered, in accordance with ethical research standards. Confidentiality and anonymity were maintained during data collection, analysis, and reporting to safeguard the privacy of the participants. Ethical guidelines and principles were strictly adhered to throughout the study to uphold the highest standards of ethical conduct in research. Researchers must adhere to all applicable institutional, national, and international regulations and guidelines when conducting experimental research or field studies on plants, including when collecting plant specimens.

### Antibacterial assay

#### Determination of minimum inhibitory concentration

The antibacterial activity test to determine the minimum inhibitory concentration (MIC) of a crude 80% ethanol and aqueous extract were conducted using the 96-well microtiter plate broth dilution method. The MIC of the plant extracts was determined using the protocols established by the Clinical and Laboratory Standards Institute (CLSI) guidelines [27, 28]. To prepare the stock solutions of the plant extracts, each extract was dissolved in distilled water and 5% tween 80, resulting in a concentration of 32 mg/ml for each solution. Quadruplicate wells were prepared in the 96-well microplates for each bacterial strain, with 100μl of Muller Hinton Broth (MHB) added to each well. Then, 100μl of the extract was added on the first row and two-fold serial dilutions with multichannel micropipettes were made down from row A to row H. Thus, serial dilutions ranging from 16 mg/mL to 0.125 mg/mL were prepared using Mueller-Hinton broth in the 96-well microplates, using a stock solution as the source. To obtain the active culture, the stored stock culture was sub-cultured at 37°C overnight on Mueller Hinton Agar. A few bacterial colonies were transferred from the sub-cultured plate to tubes containing sterile MHB using a sterile disposable inoculating loop. The test organism was standardized by preparing a diluted inoculum with an optical density (OD) range of 0.08 to 0.1 at 625nm, equivalent to a bacterial concentration of 1.0×10^8^ colony-forming units per milliliter (CFU/mL), following the guidelines provided by the CLSI, 2018. Test organism suspensions with a concentration of approximately 5×10^5^ CFU/mL well were prepared by diluting the standardized inoculum in MHB. Within 15 minutes of standardization, 100 μl of the bacterial test suspensions (5×105 CFU/mL) were added to each well of the microtiter plate, except for the sterility control (column 12). The plate was then incubated at 37°C for 18–24 hours. After incubation, a 40 μl solution of 0.4 mg/mL 2,3,5-Triphenyltetrazolium chloride (TTC) was added to each well as an indicator of microbial growth and incubated at 37°C for 30 minutes. The MIC values were determined by visually observing the presence or absence of pink color using magnifying instruments after the 30-minute incubation period. The MIC was recorded as the lowest concentration of each extract that did not display visible pink color, indicating no microbial growth. The MIC values were determined by conducting quadruplicate measurements. For each strain, a sterility control well and a growth control well were included in the study. To assess the bacterial sensitivity, parallel experiments were carried out using ciprofloxacin as a positive control, starting at a concentration of 10 μg/mL in sterile water (range of 10 μg/mL to 0.078125 μg/mL). Additionally, a negative control experiment was conducted using distilled water and 5% tween 80.

#### Determination of minimum bactericidal concentration

The determination of the minimum bactericidal concentration (MBC) was conducted following established protocols [27, 28]. In order to determine the MBC, streaking was performed on media plates by taking 5μl liquid part from each MIC well plate that showed no growth. The subcultured (inoculated) plates were then incubated under suitable conditions at 37°C for 18–24 hours. The growth of bacteria corresponding to different extract concentrations was meticulously examined. The plant extract concentration at which bacterial growth on freshly inoculated agar plates was totally inhibited was identified as the MBC. To ensure robust results, all experiments were conducted in triplicate [29].

### Phytochemical screening

Phytochemical screening was conducted on both ethanol and aqueous extracts using the methods outlined by [26, 30–32] to determine the presence of secondary metabolites. Preliminary phytochemical screening of the *S*. *somalense*, *V*. *sinaiticum*, *R*. *nervosus*, *W*. *somnifera* and *C*. *aurea* leaf aqueous extracts were tested for steroid, alkaloid, flavonoid, saponin, tannin, phenol, cardiac glycosides, terpenoid, coumarin, anthraquinine and anthocyanins. The selection of these metabolites was based on their superior potency in terms of antibacterial activity. The results were denoted as (+) for the presence and (-) for the absence of phytochemicals.

#### Test for steroid

In the phytochemical screening for steroids, two tests were conducted. In the first test, known as the Salkowski test, 2 mL of chloroform and a concentration of H_2_SO_4_ were added to 5 mL of the aqueous plant extract. A positive result was indicated by the presence of a red color in the lower chloroform layer [33]. In the second test, a mixture was prepared by combining 5 ml of chloroform and 5 ml of H_2_SO_4_ with 500 μl of the plant extract. A positive test was observed when the color changed from violet to blue or green, or when a ring of blue-green appeared. Furthermore, if the upper layer exhibited a change from yellow to green fluorescence in the H_2_SO_4_ log, it also indicated a positive result [26].

#### Test for alkaloid

In the test for alkaloids, two tests were performed. In the first test, known as Dragendroff’s test, approximately 1 ml of the extract was placed in a test tube, and a few drops of Dragendroff’s reagent, a commonly used alkaloid detection reagent, were added carefully to the test tube [26, 31, 34]. The presence of alkaloids was determined by the formation of a reddish-brown precipitate. In the second test, known as Wagner’s test, 1 ml of the extract was treated with a few drops of Wagner’s reagent, a solution of iodine in potassium iodide [26]. Again, the presence of alkaloids was indicated by the occurrence of a reddish-brown precipitate.

#### Test for flavonoids

In the phytochemical screening for flavonoids, two tests were conducted. In the first test, known as the alkaline reagent test, 2 ml of the plant extract was treated with 5 drops of dilute sodium hydroxide (NaOH), followed by the addition of diluted hydrochloric acid (HCl). The presence of flavonoids was indicated by the observed color change during the test, specifically when the yellow solution treated with NaOH turned colorless upon the addition of dilute HCl [35]. In the second test, the plant extract sample was mixed with a 10% ammonium hydroxide solution in a test tube. A positive result for flavonoids was indicated by the presence of yellow fluorescence [31].

#### Test for saponins

To detect the presence of saponins, 3 ml of the extract was vigorously shaken with 3 ml of distilled water in a test tube [26, 36, 37]. The formation of foam or froth upon shaking was used as an indicator of a positive result.

#### Test for tannin and phenol

In the test for tannin and phenol, 2 ml of FeCl_3_ solution was added to 1 ml of the plant extract. A positive result is indicated by the presence of a black or blue-green color [38].

### Test for tannin

2 ml of the tested plant extract was stirred with 3 ml of distilled water, followed by the addition of five drops of ferric chloride (FeCl). A positive test for tannins was indicated by the formation of a dark blue precipitate [37, 39].

#### Test for phenol

A few drops of dilute iodine solution were added to 1 ml of the plant extract. A positive test for phenols was indicated by the transient appearance of a red color [31, 40].

#### Test for cardiac glycocide

Keller-Killiani test was employed. A mixture was prepared by combining 1 ml of the plant extract, 1.5 ml of glacial acetic acid, and 1 drop of 5% FeCl_3_, followed by the addition of a concentrated solution of H_2_SO_4_ along the side of the test tube. The presence of a blue color in the layer of the glacial acetic acid solution indicated a positive test for cardiac glycosides [26, 38].

#### Test for terpenoid

In the method for testing terpenoids, approximately 5 ml of the plant extract was added to a mixture of 3 ml of chloroform and 2 ml of concentrated sulfuric acid (H_2_SO_4_). A positive test for terpenoids was indicated by the observation of a grey or reddish-brown color [11].

#### Test for coumarin

A NaOH test was performed by adding 10% NaOH and chloroform to the plant extract. A positive test for coumarin was indicated by the presence of a yellow color [41].

#### Test for anthraquinone

In the test for ***anthraquinone***, the Borntrager’s test was employed. A mixture was prepared by combining 10 ml of 10% ammonium solution with a few ml of the filtrate, followed by vigorous shaking for 30 seconds The observation of a pink violet or red color indicated a positive test result for anthraguinine [30, 42, 43].

#### Test for anthocyanins

In the method for testing anthocyanins, the HCL test was conducted by combining 2 ml of the plant extract with 2 ml of 2N HCL, followed by the addition of a few ml of ammonia. A positive test for anthocyanins was indicated by the transformation of the pink-red solution into a blue-violet color upon the addition of ammonia [31, 44].

## Results

### Antibacterial activity

The antibacterial activity of the five plant leaf extracts (S. *somalense*, *V*. *sinaiticum*, *R*. *nervosus*, *W*. *somnifera*, and *C*. *aurea*) evaluated against the selected bacterial strains showed different positive results. The MIC results (in mg/mL) indicate the lowest concentration of each plant extract at which visible growth of the bacteria was not observed. The antibacterial activity of extracts were screened against gram positive strains *(S*. *aureus*, *S*. *agalactiae*, *S*. *epidermidis*, *E*. *faecalis)* and gram negative bacterial strains (*P*. *mirabilis*, *S*. *typhimurium*, *K*. *pneumoniae*, *E*. *coli* and *S*. *flexner* and results obtained are summarized in Table 2.

**Table 2:** Antibacterial activity of 80% ethanol and aqueous leaf extracts against pathogenic organisms.

	Bacterial Strains	Plant extracts along with MIC and MBC result in mg/ml (Mean ± SD)										Cipro (μg/mL)	MBC
		S1		V2		R3		W4		C5			
		Ethanol	Aqueous	Ethanol	Aqueous	Ethanol	Aqueous	Ethanol	Aqueous	Ethanol	Aqueous		
**Gram-positive bacteria strains**	*Staphylococcus aureus* (ATCC 25923)	5 ±2.0	12±4.62	4±0.0	16±0.0	8±0.0	16±0.0	2±0.0	2±0.0	10±4.0	16±0.0	0.625±0.0	>16
	*Streptococcus agalactiae* (ATCC 12386)	1.5±0.58	16±0.0	4±0.0	16±0.0	16±0.0	16±0.0	3±1.15	4±0.0	16±0.0	16±0.0	0.625±0.0	>16
	*Staphylococcus epidermidis* (ATCC 12228)	12±4.62	16±0.0	12±4.62	16±0.0	8±0.0	16±0.0	3±1.15	4±0.0	16±0.0	16±0.0	0.625±0.0	>16
	*Enterococcus faecalis* (ATCC 29212)	4±0.0	16±0.0	4±0.0	16±0.0	16±0.0	16±0.0	3.5±1.0	4±0.0	16±0.0	16±0.0	1.25±0.0	>16
**Gram-negative bacteria strains**	*Proteus mirabilis* (ATCC 35659)	6±2.31	16±0.0	7±2.0	16±0.0	16±0.0	16±0.0	16±0.0	16±0.0	8±0.0	16±0.0	0.078±0.0	>16
	*Salmonella typhimurium* (ATCC 13311)	4±0.0	16±0.0	4±0.0	8±0.0	16±0.0	8±0.0	8±0.0	8±0.0	10±4.0	16±0.0	0.469±0.0	>16
	*Klebsiella pneumoniae* (ATCC 700603)	3±1.15	16±0.0	5±2.0	12±4.62	8±0.0	12±4.6	8±0.0	16±0.0	3±1.15	12±4.62	0.469±0.0	>16
	*Escherichia coli* (ATCC 25922)	3.5±1.0	12±4.62	5±2.0	16±0.0	12±4.62	16±0.0	2±0.0	16±0.0	2±0.0	16±0.0	0.352±0.0	>16
	*Shigella flexneri* (ATCC 12022)	8±0.0	8±0.0	3±1.15	4±0.0	16±0.0	16±0.0	4±0.0	3±1.15	12±4.62	16±0.0	1.250±0.0	>16

Where, S1: *Solanum somalense* Franchet. (Solanaceae), V2: *Verbascum sinaiticum* Benth. (Scrophulariaceae), R3: *Rumex nervosus* Vahl (Polygonaceae), W4: *Withania somnifera* (L.) Dunal (Solanaceae), C5: *Calpurnia aurea* (Ait.) Benth. (Fabaceae), Cipro: Ciprofloxacin. The extracts were diluted in a series of steps, starting with a concentration of 16 mg/ml and ending with a concentration of 0.125 mg/mL. Ciprofloxacin was used as a positive control, starting at a concentration of 10 μg/mL and diluting down to 0.078125 μg/mL. The comprehensive raw data supporting this study can be found in the Supporting Information section (S1 File).

**Fig 1:**
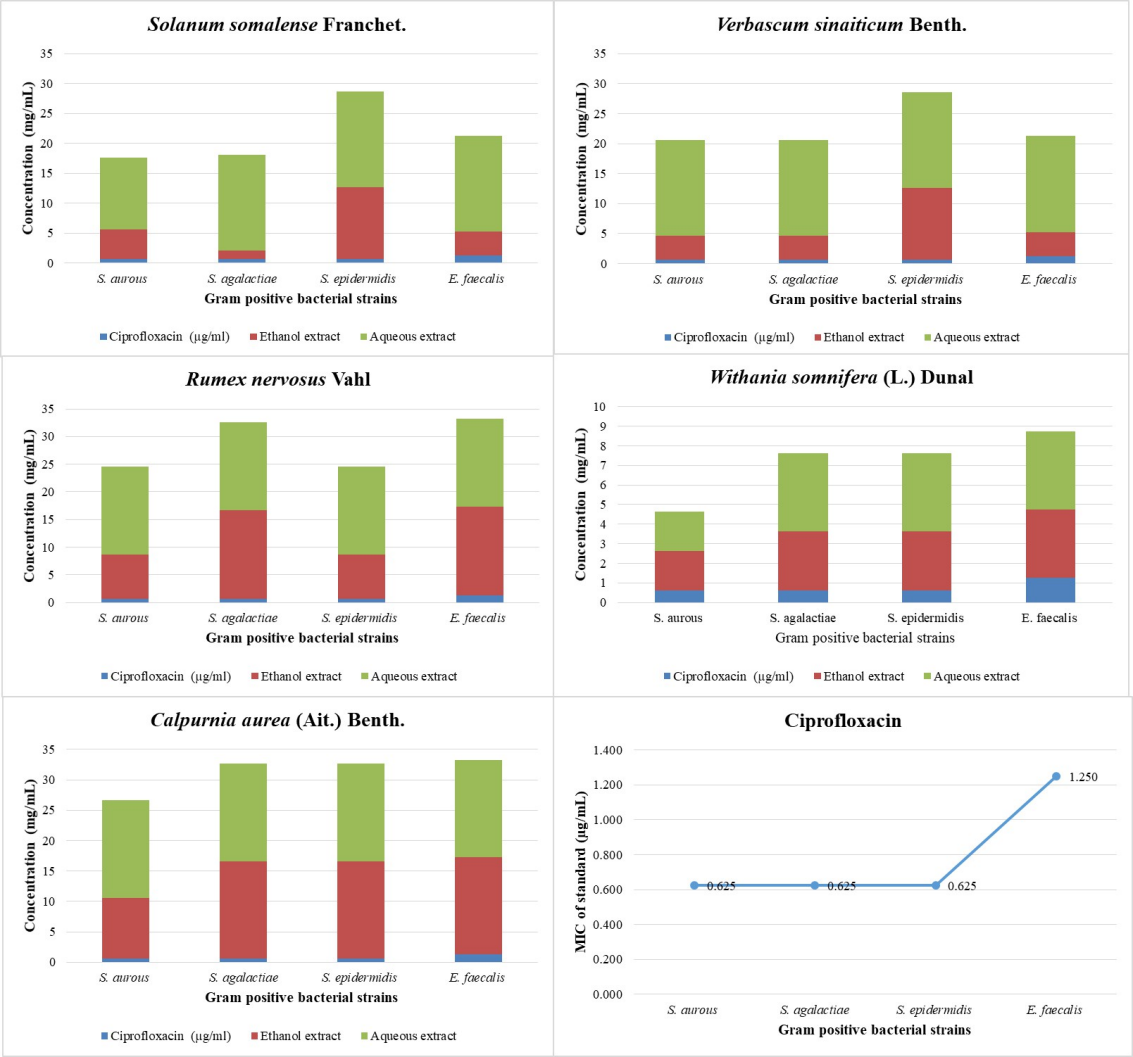
MIC of selected plant extracts against gram positive bacterial strains.

**Fig 2:**
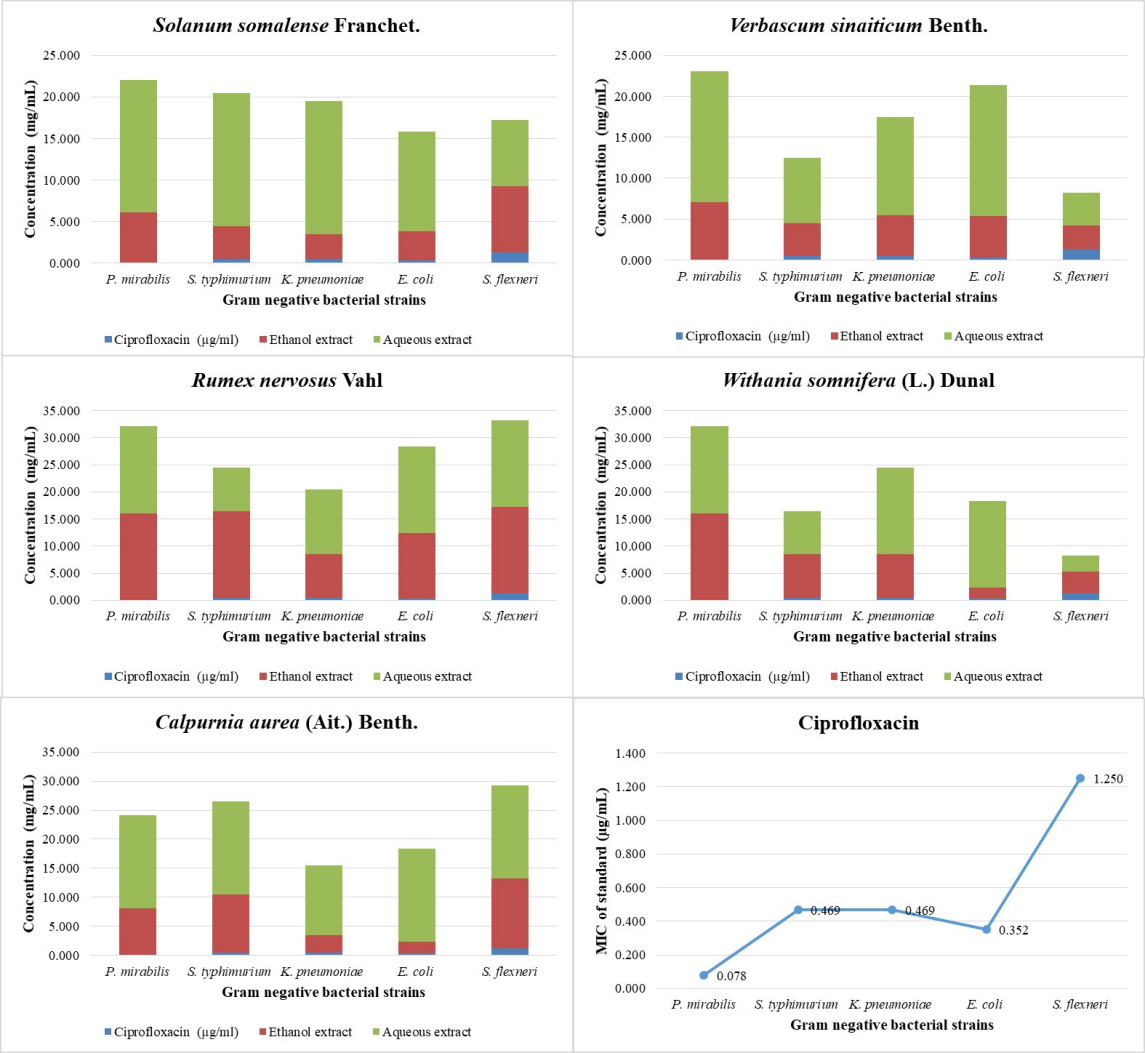
MIC of selected plant extracts against gram negative bacterial strains.

The ethanolic extract of *S*. *somalense* exhibited significant antibacterial activity against *S*. *agalactiae*, *K*. *pneumoniae*, and *E*. *coli*, with MICs of 1.5 mg/mL, 3 mg/mL, and 3.5 mg/mL, respectively. Similarly, the ethanolic extract of *W*. *somnifera* showed activity against *S*. *aureus* and *E*. *coli*, with an MIC of 2 mg/mL. Additionally, the ethanolic extract of *C*. *aurea* demonstrated activity against *E*. *coli* and *K*. *pneumoniae*, with MICs of 3 mg/mL and 3.5 mg/mL, respectively (Figs 1 & 2).

Ciprofloxacin, a commonly used antibiotic used as a positive control in the study, exhibited MIC values ranging from 0.078125 μg/mL to 1.25 μg/mL against the tested bacterial strains. This indicates the potency of ciprofloxacin in inhibiting bacterial growth at relatively low concentrations compared to the plant extracts (Fig 2).

For the gram-positive bacteria strains, the aqueous extract of *V*. *sinaiticum* exhibited lower MIC values compared to the ethanol extract. This suggests that the aqueous extract of V2 is more effective at MIC of 4 mg/mL in inhibiting the growth of *S*. *aureus*, *S*. *agalactiae*, and *E*. *faecalis*. On the other hand, the MIC values of the ethanol extract of S. *somalense*, *R*. *nervosus*, and *W*. *somnifera* were relatively higher, indicating less potent antibacterial activity against these Gram-positive bacteria strains. Ciprofloxacin, the positive control, displayed lower MIC values, indicating its strong inhibitory effect on these bacteria. Among the Gram-negative bacteria strains, the aqueous extract of *V*. *sinaiticum* again demonstrated lower MIC values compared to the ethanol extract. This suggests its stronger antibacterial activity against *P*. *mirabilis*, *S*. *typhimurium*, *K*. *pneumoniae*, *E*. *coli*, and *S*. *flexneri*. The aqueous extract of *C*. *aurea* also showed effective activity against *K*. *pneumoniae*, and E. coli with MIC of 3 mg/mL and 2 mg/mL respectively.

The MBC values were determined to assess the bactericidal effect of the plant extracts. However, the results indicated that none of the tested plant extracts exhibited bactericidal activity at the MIC tested concentrations. This implies that the concentrations of the plant extracts that inhibited bacterial growth were not sufficient to completely kill the bacteria.

### Phytochemical screening

Phytochemical screening of ethanolic extracts of five MPs (*S*. *somalense*, *V*. *sinaiticum*, *R*. *nervosus*, *W*. *somnifera*, and *C*. *aurea*) was carried out in order to identify either the presence or absence of secondary metabolites which are steroid, alkaloid, flavonoid, saponin, tannin, phenol, cardiac glycosides, terpenoid, coumarin, anthraguinine and anthocyanins as shown in Table 3. All tested extracts exhibited positive results for steroids in the Salkowski test, which involved the preparation of a mixture comprising 5 mL of chloroform, 5 mL of H_2_SO_4_, and 500 μl of the plant extract. A positive test was indicated by the formation of a blue-green ring. The phytochemical screening of *S*. *somalense* demonstrated positive results for steroids, alkaloids, flavonoids, saponins, and terpenoids. *V*. *sinaiticum* and *C*. *aurea* exhibited the presence of all chemical compounds tested, except for Anthraquinones, which was only detected in *V*. *sinaiticum*. Anthocyanins were not detected in any of the tests conducted in this study. Tannins and phenolic compounds were found to be abundant in the tested samples, potentially in the form of tannins, as indicated by the formation of a dark precipitate upon treatment with a ferric chloride solution. However, *S*. *somalense* did not show the formation of a dark precipitate in the ferric chloride test. Cardiac glycosides, as detected by the Keller-Killiani test, and coumarins, as indicated by the NaOH test, were exclusively present in *V*. *sinaiticum* and *C*. *aurea*. Alkaloids, steroids, and flavonoids were found to be relatively common in the ethanolic extracts, indicating their presence in significant amounts. On the other hand, anthraquinones and anthocyanins were observed to be the least abundant compounds in the tested extracts.

**Table 3:** Phytochemical screening tested medicinal plants.

Phytochemical Components	Test	Plant Extracts				
		S1	V2	R3	W4	C5
Steroids	Salkowski test	-	+	-	+	-
	Chloroform + H_2_SO_4_	+	+	+	+	+
Alkaloids	Dragendroff’s test	-	-	-	-	-
	Wagner’s test	+	+	-	-	+
Flavonoids	Alkaline reagent test	+	+	+	-	+
	Alkaline reagent test using 10% ammonium hydroxide sol.	-	+	-	-	-
Saponin	Foam test	+	+	+	-	+
Tannins and Phenolic	FeCl_3_ test	-	+	+	+	+
Tannins	Ferric chloride (FeCl) test	-	+	+	+	+
Phenolic	Iodine test	-	+	+	+	+
Cardiac Glycosides	Keller-Killiani test	-	+	-	-	+
Terpinoides	Salkowski test	+	+	+	-	+
Coumarins	NaOH test	-	+	-	-	+
Anthraquinones	Borntrager’s test	-	+	-	-	-
Anthocyanins	HCL test	-	-	-	-	-

+ = positive;— = negative; S1: *S*. *somalense*, V2: *V*. *sinaiticum*, R3: *R*. *nervosus*, W4: *W*. *somnifera*, C5: *C*. *aurea*

## Discussion

The results of the present study showed that the ethanolic extracts of the plants that had higher informant consensus and fidelity level values (*S*. *somalense*, *W*. *somnifera* and *C*. *aurea)* exhibited antibacterial activity against the selected bacterial strains. The ethanolic extract of *S*. *somalense* demonstrated antibacterial activity against *S*. *agalactiae*, *K*. *pneumoniae*, and *E*. *coli*, with MICs of 1.5 mg/mL, 3 mg/mL, and 3.5 mg/mL, respectively. This indicates the potential of *S*. *somalense* as a source of antibacterial compounds. The ethanolic extract of *W*. *somnifera* also showed activity against *S*. *aureus* and *E*. *coli*, with an MIC of 2 mg/mL. This suggests that *W*. *somnifera* has antibacterial properties against gram-positive and gram-negative bacteria. Likewise, the MICs of 3 mg/mL and 3.5 mg/mL were recorded for the ethanolic extract of *C*. *aurea* against *E*. *coli* and *K*. *pneumoniae*, respectively. This indicates the potential of *C*. *aurea* as an antibacterial agent.

Numerous studies [12, 13, 15, 17, 19, 45, 46] conducted in various countries have reported a significant success rate in antibacterial activity tests, aligned with the ethnomedicinal background. A synthesis of findings from various studies reveals a consistent pattern of antibacterial efficacy across a diverse range of plants, despite differences in phytochemical composition and geographical origins. Notably, research by Ermias Lulekal et al. [19] highlights that 74% of ethanol extracts from various plants exhibited antimicrobial activity, with *E*. *schimperi*, *O*. *lamiifolium*, and *R*. *steudneri* showing broad-spectrum activity. This aligns with the findings of Rawat & Bisht [47], who reported the potent antibacterial activity of the methanolic extract of *W*. *somnifera* against gram-positive clinical isolates. Similarly, Dharajiya et al. [46] found that methanol extracts of *W*. *somnifera* were highly effective against *E*. *coli* and *B*. *cereus*. Al Yahya et al. [48] contributed to this body of knowledge by demonstrating the significant antimicrobial activity of methanol and n-hexane extracts of *R*. *nervosus*. Dessie Belay et al. [14] extended this understanding by revealing the antimicrobial and antioxidant activities of solvent fractions of *C*. *aurea* leaves. Furthermore, Gebremedhin Romha et al. [16] and Shemsu Umer et al. [17] both observed promising antibacterial activities in methanol and chloroform extracts of *C*. *macrostachyus* Del., with *Calpurnia aurea* extracts exhibiting the highest zone of inhibition against *S*. *aureus*. Also, Solomon Berhanu [49] found that extracts of *C*. *aurea* were effective against both *E*. *coli* and *S*. *aureus*, contributing to the growing evidence of the plant’s antibacterial properties. These studies predominantly align with our research in terms of the ethnomedicinal plants examined, although there are variations in species across different regions. The significant success rates in antibacterial activity tests reported in these studies corroborate the ethnomedicinal use of these plants, reinforcing the validity of traditional knowledge in identifying potential antibacterial agents.

Overall, the results of the present study suggest that the ethanolic extracts of *S*. *somalense*, *W*. *somnifera*, and *C*. *aurea* have potential as antibacterial agents. The methanol extract of *W*. *somnifera* stem showed the maximum antibacterial activity against *E*. *coli*. The range of MIC of extracts recorded was 3.125 to 50 mg/mL [46], with the lowest MIC value of 3.125 mg/mL being recorded for methanol extract against *E*. *coli*. The paper reported the presence of alkaloids, tannins, saponins, cardiac glycosides, steroids, phenols, and flavonoids in the methanol and aqueous extracts of the stem of *W*. *somnifera*. However, glycosides and sterol were absent in the hexane extract, and cardiac glycoside was absent in the ethyl acetate extract. Overall, the aqueous extracts of *V*. *sinaiticum* and *C*. *aurea* demonstrated better antibacterial activity against both gram-positive and gram-negative bacteria strains compared to the ethanol extracts. However, none of the tested plant extracts exhibited bactericidal effects at the tested MIC concentrations. This suggests that the extracts may inhibit the growth of bacteria but do not completely kill them.

Phytochemical screening of the ethanolic extracts of the five medicinal plants revealed the presence of various secondary metabolites. All tested extracts exhibited positive results for steroids in the Salkowski test. This indicates the presence of steroids in all the plant extracts. *S*. *somalense* showed positive results for steroids, alkaloids, flavonoids, saponins, and terpenoids. This suggests that *S*. *somalense* contains a diverse range of bioactive compounds. *V*. *sinaiticum* and *C*. *aurea* exhibited the presence of most chemical compounds tested, except for anthraquinones, which were only detected in *V*. *sinaiticum*. Anthocyanins were not detected in any of the tested extracts. The absence of Anthocyanins in all the tested extracts may be attributed to several factors. Anthocyanins are water-soluble pigments responsible for the vibrant red, purple, or blue colors in various fruits, vegetables, and flowers [50]. They are commonly found in plants belonging to the families Rosaceae, Solanaceae, and Ericaceae, among others, which include fruits like strawberries, blueberries, and cherries. *S*. *somalense* did not show the formation of a dark precipitate in the ferric chloride test, indicating a difference in the presence of tannins compared to other plants. Cardiac glycosides and coumarins were exclusively present in *V*. *sinaiticum* and *C*. *aurea*. Alkaloids, steroids, and flavonoids were relatively common in the ethanolic extracts, indicating their significant presence. Indeed, understanding the precise modes of action can provide insights into how these compounds interact with bacterial cells, potentially leading to the development of more effective antimicrobial agents.

The results obtained from various studies further support the potential of these plants as sources of antibacterial agents and highlight their diverse biological activities. Ethanol extracts obtained from the leaves of *V*. *sinuatum* L. exhibited strong antibacterial activity against *E*. *faecalis* and *P*. *mirabilis* [18]. This finding reinforces the potential of *V*. *sinuatum* L. as an effective antibacterial agent. Another species of Verbascum, *V*. *lasianthum* Boiss. ex Bentham, was investigated for its biological activity and element content by [15]. The study revealed that different concentrations of *V*. *lasianthum* extracts possess antibacterial activity, along with antioxidant and cytotoxic properties. This further supports the medicinal potential of Verbascum species.

In addition to antibacterial activity, *V*. *sinaiticum* has also been explored for its wound healing and antioxidant properties. The hydroalcoholic leaf extract of *V*. *sinaiticum* in excision and incision wound models [12]. The results demonstrated significant wound healing efficacy, as evidenced by an enhanced wound contraction rate. This suggests that *V*. *sinaiticum* extracts have potential applications in wound healing therapies. Furthermore, *V*. *sinaiticum* has been found to possess analgesic and anti-inflammatory properties. 80% methanol root extract of *V*. *sinaiticum* exhibited peripheral and central analgesic effects, along with anti-inflammatory activity [13]. These beneficial effects could be attributed to the presence of phytochemicals such as alkaloids, tannins, flavonoids, phenols, steroids, and glycosides.

Regarding *C*. *aurea*, it has also demonstrated antimicrobial activity against various bacterial strains. The activity of *V*. *sinaiticum* and *C*. *aurea* against *S*. *aureus* and *E*. *coli*, with different minimum inhibitory concentrations [45]. This indicates the potential of *C*. *aurea* as an effective antimicrobial agent. The ethanol extract of *C*. *aurea* leaves and bark and found significant antimicrobial activity against *E*. *coli* and *S*. *aureus* [16, 49]. The study also identified the presence of various phytochemicals, including phenolic compounds, saponins, flavonoids, alkaloids, and others in the plant extracts. These compounds contribute to the antibacterial potential of *C*. *aurea*.

Moreover, the antimicrobial activity of the 80% methanol extract of *C*. *aurea* leaves against a range of organisms, including *Salmonella typhimurium*, *Shigella* species, *P*. *aeruginosa*, *S*. *aureus*, and *E*. *coli* [17]. The methanol extract of *C*. *aurea* exhibited moderate inhibition of bacterial growth, with the highest activity observed against *E*. *coli*. Additionally, the antimicrobial effects of 95% methanol extracts of *C*. *aurea* leaves [14]. Their study revealed significant antimicrobial activity of the ethyl acetate and dichloromethane fractions of the leaves against pathogenic bacteria, including *S*. *aureus*, *E*. *coli*, *E*. *faecalis*, and *K*. *pneumoniae*. These findings provide further support for the potential of *C*. *aurea* as a valuable source of antibacterial compounds.

The positive outcomes observed in the antibacterial activity tests coupled with the presence of bioactive compounds in these extracts establish a promising foundation for pharmaceutical exploration. For instance, the correlation between the detected bioactive compounds, such as alkaloids, flavonoids, and steroids in *S*. *somalense* aligns with its observed antibacterial potential against specific bacterial strains, thereby validating its traditional use in ethnomedicine. Similarly, the presence of diverse chemical constituents, such as tannins and phenols, in *W*. *somnifera* and *C*. *aurea* echoes their noted antibacterial activity against different bacteria, corroborating their historical medicinal applications. While our study suggests the possibility of novel antimicrobial agent development from these plants, further investigations are crucial to decipher the precise mechanisms of action and potential synergies among these bioactive compounds. This comprehensive understanding bridges the traditional knowledge of these medicinal plants with our scientific findings, and may pave the way for the development of novel antimicrobial agents with potential pharmacological benefits.

## Conclusion

The current study demonstrated the antibacterial activity of the selected medicinal plant extracts against gram-positive and gram-negative bacterial strains. The MIC results highlighted the inhibitory concentrations of the extracts, while the MBC results indicate the absence of bactericidal effects at the tested concentrations. Accordingly, the extracts of *S*. *somalense*, *W*. *somnifera* and *C*. *aurea* exhibit varying degrees of antimicrobial activities at MIC range from 1.5–3.5 mg/mL against pathogenic microbes namely *S*. *aureus*, *S*. *agalactiae*, *K*. *pneumoniae* and *E*. *coli*. The phytochemical screening of the selected medicinal plants revealed diverse profiles of phytochemical components. Insights of these findings can stimulate further in-depth investigations to maximize the advantages that could be provided by these plant extracts for regular use as effective antimicrobial agents. The results confirm that the people of the study area have developed indigenous knowledge about the efficacies of the tested species through generations of repeated use. The research tested not only the plants but also the indigenous medical practice of the people of Habru District.

## Supporting information

S1 File Raw data for minimum inhibitory concentration (MIC) determination. (XLS)
